# Supplementary material for: Whole transcriptome sequencing and biomineralization gene architecture associated with cultured pearl quality traits in the pearl oyster, Pinctada margaritifera
Source: BMC Genomics. 2019 Feb 6;20:111. doi: 10.1186/s12864-019-5443-5 (PMC6366105; doi:10.1186/s12864-019-5443-5)
Supplement: Supplementary file 1 — Figure S1. Summarized REVIGO semantic plot for gene ontology enrichment analysis. Figure S2. Relative gene expression for biomineralization genes analysed by qPCR in the pearl sac of P. margaritifera. Values are expressed as means of relative expression ± standard deviation. Asterisks indicate significant differences (Wilcoxon test, p-value < 0.01). Table S1. P. margaritifera individuals used for the transcriptome assembly. NA = Not identified. Table S2. Complete list and statistics on differentially expressed genes and their annotation. Table S3. Set of forward and reverse primers used for the biomineralization gene expression (real-time PCR) analysis in Pinctada margaritifera. (DOCX 795 kb) [file 12864_2019_5443_MOESM1_ESM.docx]

Additional file 1

Whole transcriptome sequencing and biomineralization gene architecture associated with cultured pearl quality traits in the pearl oyster, *Pinctada margaritifera*

Le Luyer J.^1^*, Auffret P. ^1^, Quillien V. ^1^, Leclerc N. ^1^, Reisser C.^1^, Vidal-Dupiol J.^2,1^, Ky C.-L.^1,2^*

^1^ IFREMER, UMR 241 Ecosystèmes Insulaires Océaniens, Centre Ifremer du Pacifique, BP 49, 98719 Tahiti, Polynésie française

^2^ IFREMER, IHPE UMR 5244, Univ. Perpignan Via Domitia, CNRS, Univ. Montpellier, F-34095 Montpellier, France

***Corresponding authors:** jeremy.le.luyer@ifremer.fr; chinky@ifremer.fr

Figure S1.


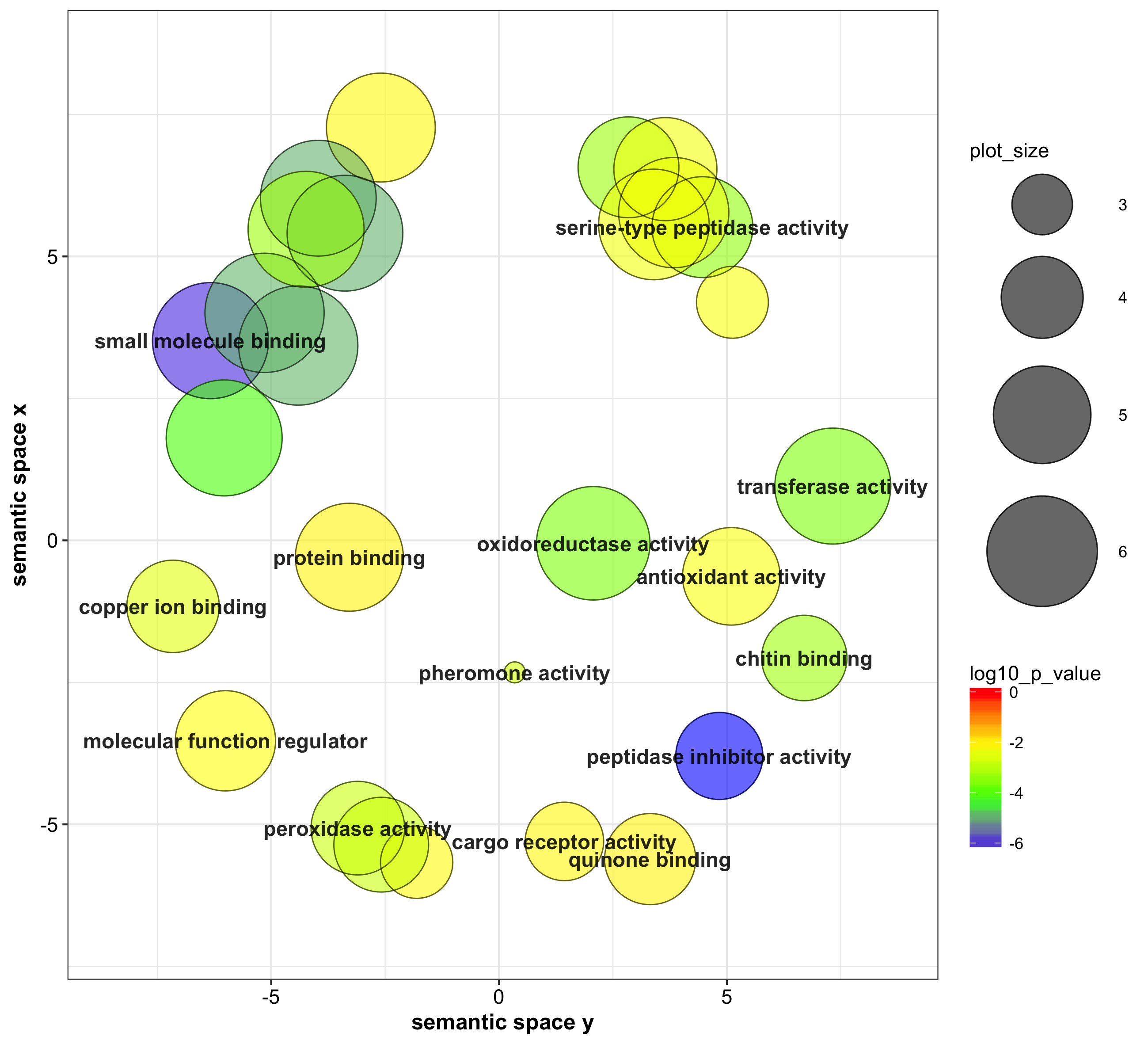


Figure S1: Summarized REVIGO semantic plot for gene ontology enrichment analysis

Figure S2.

Figure S2: Relative gene expression for biomineralization genes analysed by qPCR in the pearl sac of *P. margaritifera*. Values are expressed as means of relative expression ± standard deviation. Asterisks indicate significant differences (Wilcoxon test, *p*-value < 0.01).

Table S1.

Table S1: *P. margaritifera* individuals used for the transcriptome assembly. NA = Not identified.

| **Individuals** | **Tissue** | **Sex / colour / age** | **Nb. Raw PE reads (x10^6^)** | **Nb. Filtered PE reads (x10^6^)** |
| --- | --- | --- | --- | --- |
| HI.4112.001.D707---D506.X4 | mantle | NA / orange / adult | 36.40 | 32.78 |
| HI.4112.001.D707---D507.X6 | mantle | NA / black/ adult | 33.87 | 30.60 |
| HI.4112.001.D708---D504.X13 | mantle | NA/ albinos / adult | 32.62 | 29.52 |
| HI.4112.002.D710---D502.X19 | mantle | NA / red / adult | 33.15 | 30.23 |
| HI.4112.002.D710---D503.X21 | mantle | NA / yellow / adult | 30.87 | 28.40 |
| HI.4112.002.D711---D501.X30 | mantle | NA / green / adult | 37.62 | 34.67 |
| SRR1039667_sample123 | gonad | Male / NA / adult | 41.16 | 37.00 |
| SRR1041217_sample144 | gonad | Female / NA / adult | 38.57 | 36.92 |
| HI.4274.008.Index_15.TB2 | whole | NA / black/ juvenile | 22.12 | 19.33 |
| HI.4274.008.Index_7.TB9 | whole | NA / black / juvenile | 40.21 | 31.83 |

Table S2.

Table S2: Complete list and statistics on differentially expressed genes and their annotation.

| **Gene** | **Log2FC** | **FDR** | **Uniprot Accession** | **Uniprot**  **ID** | ***P. fucata*** | ***C. gigas*** | **Best hit** |
| --- | --- | --- | --- | --- | --- | --- | --- |
| TRINITY_DN30164_c0_g1_i1 | -5.41 | 4.42E-05 |  |  | pfu_aug1.0_1659.1_08045.t1 |  | pfu_aug1.0_1659.1_08045.t1 |
| TRINITY_DN44125_c0_g1_i1 | 1.99 | 5.97E-03 |  |  | pfu_aug1.0_48073.1_63315.t1 | XP_019922388.1 | pfu_aug1.0_48073.1_63315.t1 |
| TRINITY_DN46643_c0_g1_i1 | -4.23 | 9.77E-08 |  |  |  |  |  |
| TRINITY_DN48625_c0_g1_i1 | -3.97 | 2.49E-04 |  |  |  |  |  |
| TRINITY_DN49174_c0_g1_i2 | -1.23 | 2.55E-03 | P17809 | GTR1_MOUSE | pfu_aug1.0_28228.1_47878.t1 | XP_011439053.1 | XP_011439053.1 |
| TRINITY_DN50008_c0_g1_i2 | -7.51 | 2.99E-05 | P18428 | LBP_HUMAN | pfu_aug1.0_5178.1_30885.t1 |  | pfu_aug1.0_5178.1_30885.t1 |
| TRINITY_DN52560_c0_g1_i2 | -4.25 | 9.42E-10 |  |  | pfu_aug1.0_7934.1_45765.t1 |  | pfu_aug1.0_7934.1_45765.t1 |
| TRINITY_DN53030_c0_g1_i2 | -6.59 | 1.32E-20 |  |  | pfu_aug1.0_5514.1_23808.t1 | XP_011434249.1 | pfu_aug1.0_5514.1_23808.t1 |
| TRINITY_DN53306_c0_g1_i1 | -2.33 | 9.40E-06 |  |  | pfu_aug1.0_25579.1_69718.t1 | XP_011444699.1 | pfu_aug1.0_25579.1_69718.t1 |
| TRINITY_DN53585_c0_g1_i1 | 1.41 | 9.92E-03 |  |  | pfu_aug1.0_4062.1_01612.t1 | XP_011452003.1 | pfu_aug1.0_4062.1_01612.t1 |
| TRINITY_DN53977_c0_g1_i1 | -1.31 | 3.15E-05 | Q7YT83 | TX31_CONTE | pfu_aug1.0_163.1_36325.t1 | XP_011449941.1 | XP_011449941.1 |
| TRINITY_DN54607_c0_g1_i1 | -1.93 | 7.45E-03 | P04111 | SPE2C_STRPU | pfu_aug1.0_49599.1_56078.t1 | XP_011449110.1 | pfu_aug1.0_49599.1_56078.t1 |
| TRINITY_DN55617_c0_g1_i1 | -2.52 | 3.94E-03 |  |  |  |  |  |
| TRINITY_DN55726_c0_g1_i1 | -4.61 | 7.25E-03 |  |  | pfu_aug1.0_507.1_36562.t1 |  | pfu_aug1.0_507.1_36562.t1 |
| TRINITY_DN56049_c0_g1_i2 | -2.4 | 3.40E-03 |  |  |  |  |  |
| TRINITY_DN56342_c0_g1_i3 | -4.61 | 6.00E-08 |  |  | pfu_aug1.0_15024.1_25368.t1 | XP_011429496.2 | XP_011429496.2 |
| TRINITY_DN56585_c0_g1_i3 | -4.4 | 2.11E-04 |  |  | pfu_aug1.0_7358.1_67675.t1 | XP_019918517.1 | XP_019918517.1 |
| TRINITY_DN56658_c0_g1_i1 | -2.74 | 2.80E-06 |  |  |  | XP_011455360.1 | XP_011455360.1 |
| TRINITY_DN56698_c0_g2_i1 | -3.4 | 8.95E-03 | Q9ESN4 | C1QL3_MOUSE | pfu_aug1.0_3850.1_37727.t1 | XP_019925362.1 | pfu_aug1.0_3850.1_37727.t1 |
| TRINITY_DN56873_c0_g1_i1 | -5.14 | 1.11E-04 | P86952 | TYRO_PINMA | pfu_aug1.0_12145.1_17832.t1 | XP_011453423.1 | P86952 |
| TRINITY_DN56924_c0_g1_i2 | -5.61 | 9.34E-10 |  |  | pfu_aug1.0_7934.1_45765.t1 | XP_011412575.1 | pfu_aug1.0_7934.1_45765.t1 |
| TRINITY_DN56942_c0_g4_i1 | -4.28 | 6.92E-03 |  |  | pfu_aug1.0_5136.1_59715.t1 |  | pfu_aug1.0_5136.1_59715.t1 |
| TRINITY_DN57084_c0_g1_i1 | -3.69 | 5.85E-07 |  |  |  |  |  |
| TRINITY_DN57165_c0_g1_i2 | 2.27 | 9.90E-03 | Q8BG95 | MYPT2_MOUSE | pfu_aug1.0_11910.1_60958.t1 | XP_011424650.1 | pfu_aug1.0_11910.1_60958.t1 |
| TRINITY_DN57483_c0_g1_i3 | -3.93 | 3.40E-09 |  |  | pfu_aug1.0_10954.1_17695.t1 | XP_011448815.2 | XP_011448815.2 |
| TRINITY_DN57507_c0_g1_i3 | -5.11 | 1.04E-09 | Q06805 | TIE1_BOVIN |  | XP_011452009.1 | XP_011452009.1 |
| TRINITY_DN57689_c0_g4_i2 | -6.33 | 2.60E-11 | P62810 | WFD18_MOUSE | pfu_aug1.0_102.1_29088.t1 | XP_011453604.1 | pfu_aug1.0_102.1_29088.t1 |
| TRINITY_DN57828_c0_g1_i1 | -4.89 | 1.92E-03 | P31426 | PAL2_SOLTU | pfu_aug1.0_1058.1_29550.t1 | XP_019919705.1 | pfu_aug1.0_1058.1_29550.t1 |
| TRINITY_DN57898_c0_g1_i6 | 1.27 | 3.99E-03 | B3A0P4 | USP26_LOTGI | pfu_aug1.0_4062.1_01612.t1 | XP_011452008.1 | pfu_aug1.0_4062.1_01612.t1 |
| TRINITY_DN57906_c0_g1_i10 | -1.24 | 1.04E-05 | P47238 | PAX6_COTJA | pfu_aug1.0_406.1_65408.t1 | XP_011433289.1 | XP_011433289.1 |
| TRINITY_DN57958_c0_g2_i1 | -4.2 | 6.47E-04 | H2A0L0 | TYRO1_PINMG | pfu_aug1.0_3212.1_37533.t1 | XP_011413535.1 | H2A0L0 |
| TRINITY_DN57987_c0_g1_i1 | -4.31 | 6.15E-03 |  |  | pfu_aug1.0_3134.1_59151.t1 | XP_019926643.1 | pfu_aug1.0_3134.1_59151.t1 |
| TRINITY_DN58089_c0_g1_i2 | -1.26 | 8.37E-03 |  |  | pfu_aug1.0_17748.1_25649.t1 |  | pfu_aug1.0_17748.1_25649.t1 |
| TRINITY_DN58261_c0_g1_i1 | -2.93 | 2.29E-03 |  |  | pfu_aug1.0_20034.1_33062.t1 | XP_011428038.2 | XP_011428038.2 |
| TRINITY_DN58300_c1_g1_i1 | 5.1 | 2.38E-03 |  |  |  |  |  |
| TRINITY_DN58345_c0_g2_i2 | -1.28 | 3.39E-03 | Q4ZJM9 | C1QL4_MOUSE | pfu_aug1.0_3137.1_37511.t1 | XP_011411936.1 | pfu_aug1.0_3137.1_37511.t1 |
| TRINITY_DN58358_c0_g1_i1 | -3.34 | 2.02E-03 |  |  | pfu_aug1.0_5628.1_52578.t1 | XP_011416798.1 | pfu_aug1.0_5628.1_52578.t1 |
| TRINITY_DN58360_c0_g1_i1 | -2.52 | 4.86E-04 |  |  | pfu_aug1.0_615.1_65547.t1 |  | pfu_aug1.0_615.1_65547.t1 |
| TRINITY_DN58608_c0_g1_i2 | -7.06 | 4.42E-05 | C7G0B5 | PIF_PINFU | pfu_aug1.0_170.1_36339.t1 | XP_019922070.1 | pfu_aug1.0_170.1_36339.t1 |
| TRINITY_DN58764_c0_g1_i3 | 2 | 3.39E-03 |  |  | pfu_aug1.0_26254.1_69760.t1 | XP_011417252.1 | XP_011417252.1 |
| TRINITY_DN58992_c0_g1_i1 | -1.01 | 3.39E-03 |  |  | pfu_aug1.0_94684.1_28039.t1 |  | pfu_aug1.0_94684.1_28039.t1 |
| TRINITY_DN59022_c0_g1_i1 | -3.04 | 2.28E-03 | P05099 | MATN1_CHICK | pfu_aug1.0_107607.1_49613.t1 | XP_011432738.1 | XP_011432738.1 |
| TRINITY_DN59203_c0_g1_i3 | -2.86 | 8.28E-20 | Q5R941 | FKB14_PONAB | pfu_aug1.0_82.1_21956.t1 | XP_011415688.1 | XP_011415688.1 |
| TRINITY_DN59296_c0_g1_i2 | -2.72 | 2.28E-03 | Q7Z0T3 | TEMPT_APLCA | pfu_aug1.0_3412.1_51982.t1 | XP_011448207.1 | pfu_aug1.0_3412.1_51982.t1 |
| TRINITY_DN59301_c1_g2_i6 | -5.03 | 8.55E-16 |  |  |  |  |  |
| TRINITY_DN59368_c0_g1_i1 | -3.84 | 8.37E-07 | P00639 | DNAS1_BOVIN | pfu_aug1.0_1574.1_29764.t1 | XP_019928552.1 | pfu_aug1.0_1574.1_29764.t1 |
| TRINITY_DN59512_c0_g1_i1 | -3.68 | 1.49E-08 |  |  | pfu_aug1.0_11553.1_17768.t1 | XP_011439845.1 | pfu_aug1.0_11553.1_17768.t1 |
| TRINITY_DN59586_c0_g1_i1 | -1.13 | 8.94E-04 |  |  |  | XP_011412656.1 | XP_011412656.1 |
| TRINITY_DN59621_c0_g1_i3 | -3.76 | 5.06E-08 |  |  |  |  |  |
| TRINITY_DN59668_c0_g1_i2 | -1.72 | 7.45E-04 |  |  | pfu_aug1.0_4889.1_09005.t1 |  | pfu_aug1.0_4889.1_09005.t1 |
| TRINITY_DN59693_c5_g1_i2 | -3.26 | 2.80E-10 | P18203 | FKB1A_BOVIN | pfu_aug1.0_82.1_21957.t1 | XP_011429379.1 | XP_011429379.1 |
| TRINITY_DN60230_c0_g1_i3 | 1.34 | 9.72E-03 | Q9WU22 | PTN4_MOUSE | pfu_aug1.0_2526.1_23002.t1 | XP_011429619.1 | pfu_aug1.0_2526.1_23002.t1 |
| TRINITY_DN60258_c0_g1_i7 | -2.5 | 1.69E-06 |  |  |  |  |  |
| TRINITY_DN60330_c2_g1_i7 | -2.38 | 2.69E-05 |  |  | pfu_aug1.0_15.1_14687.t1 |  | pfu_aug1.0_15.1_14687.t1 |
| TRINITY_DN60660_c0_g1_i2 | -5.07 | 2.18E-03 | H2A0M4 | USP1_PINMG | pfu_aug1.0_12300.1_32179.t1 | XP_011456443.1 | H2A0M4 |
| TRINITY_DN60684_c1_g1_i4 | -4.7 | 8.25E-22 | H2A0L3 | ELDP2_PINMG | pfu_aug1.0_853.1_22356.t1 | XP_011437414.1 | H2A0L3 |
| TRINITY_DN60953_c0_g1_i9 | -4.37 | 2.32E-03 |  |  |  |  |  |
| TRINITY_DN60987_c0_g1_i1 | -1.32 | 2.41E-04 |  |  | pfu_aug1.0_9677.1_38951.t1 | XP_011412540.1 | pfu_aug1.0_9677.1_38951.t1 |
| TRINITY_DN60989_c9_g1_i1 | -7.01 | 3.11E-11 |  |  | pfu_aug1.0_3838.1_37722.t1 |  | pfu_aug1.0_3838.1_37722.t1 |
| TRINITY_DN60989_c9_g2_i2 | -5.64 | 1.00E-26 |  |  | pfu_aug1.0_3838.1_37722.t1 | XP_019925720.1 | pfu_aug1.0_3838.1_37722.t1 |
| TRINITY_DN61135_c6_g2_i3 | -2.58 | 1.13E-03 |  |  | pfu_aug1.0_199.1_36374.t1 | XP_011435143.1 | pfu_aug1.0_199.1_36374.t1 |
| TRINITY_DN61168_c0_g1_i1 | -6.35 | 6.18E-05 |  |  |  |  |  |
| TRINITY_DN61168_c0_g2_i1 | -6.5 | 3.53E-07 | H2A0K8 | SLP1_PINMG | pfu_aug1.0_114185.1_13709.t1 | | H2A0K8 |
| TRINITY_DN61536_c0_g1_i1 | -2.83 | 3.01E-11 |  |  | pfu_aug1.0_10577.1_53557.t1 |  | pfu_aug1.0_10577.1_53557.t1 |
| TRINITY_DN61647_c4_g2_i4 | -2.51 | 2.87E-04 | H2A0L7 | FND1_PINMG | pfu_aug1.0_241391.1_43079.t1 | XP_011414106.1 | H2A0L7 |
| TRINITY_DN61773_c2_g1_i4 | -4.97 | 8.47E-03 | H2A0K7 | MP_PINMG | pfu_aug1.0_3035.1_59110.t1 | XP_011425195.1 | H2A0K7 |
| TRINITY_DN61852_c0_g1_i2 | -4.54 | 6.00E-03 | C7G0B5 | PIF_PINFU | pfu_aug1.0_2573.1_44416.t1 | XP_011415561.1 | pfu_aug1.0_2573.1_44416.t1 |
| TRINITY_DN61891_c2_g1_i2 | -3.45 | 2.52E-04 |  |  |  |  |  |
| TRINITY_DN61894_c0_g1_i2 | -5.29 | 4.54E-05 |  |  | pfu_aug1.0_507.1_36561.t1 |  | pfu_aug1.0_507.1_36561.t1 |
| TRINITY_DN62046_c5_g3_i1 | 2.78 | 2.32E-04 | P21328 | RTJK_DROME | pfu_aug1.0_811.1_58262.t1 | XP_019920953.1 | XP_019920953.1 |
| TRINITY_DN62440_c0_g3_i8 | -2.24 | 9.03E-06 |  |  |  | XP_011423627.2 | |
| TRINITY_DN62577_c2_g1_i2 | -3.3 | 2.85E-03 |  |  | pfu_aug1.0_10099.1_31878.t1 |  | pfu_aug1.0_10099.1_31878.t1 |
| TRINITY_DN62782_c0_g1_i5 | -4.62 | 4.08E-03 |  |  |  |  |  |
| TRINITY_DN62918_c0_g1_i2 | -3.5 | 1.18E-04 |  |  |  |  |  |
| TRINITY_DN62954_c5_g1_i3 | -1.83 | 6.99E-06 |  |  | pfu_aug1.0_28039.1_04619.t1 | XP_011413496.2 | pfu_aug1.0_28039.1_04619.t1 |
| TRINITY_DN63112_c4_g1_i1 | -2.25 | 6.05E-04 |  |  |  |  |  |
| TRINITY_DN63165_c1_g2_i6 | -5.42 | 2.02E-03 | P86950 | SLP2_PINMA |  |  | P86950 |
| TRINITY_DN63306_c0_g1_i3 | -5.21 | 3.16E-08 |  |  |  |  |  |
| TRINITY_DN63385_c0_g1_i1 | -3.11 | 7.14E-04 | Q8IUA0 | WFDC8_HUMAN | pfu_aug1.0_654.1_07616.t1 | XP_011453771.1 | pfu_aug1.0_654.1_07616.t1 |
| TRINITY_DN63435_c3_g1_i14 | -1.39 | 1.72E-03 |  |  | pfu_aug1.0_51875.1_41528.t1 |  | pfu_aug1.0_51875.1_41528.t1 |
| TRINITY_DN63445_c0_g1_i5 | -6.4 | 5.62E-14 |  |  | pfu_aug1.0_15966.1_10874.t1 |  | pfu_aug1.0_15966.1_10874.t1 |
| TRINITY_DN63468_c5_g1_i2 | 4.29 | 5.05E-04 | Q6P5C5 | SMUG1_MOUSE |  | XP_011427768.1 | XP_011427768.1 |
| TRINITY_DN63878_c0_g1_i2 | -3.86 | 1.33E-07 | P80003 | PA2A2_HELSU | pfu_aug1.0_1966.1_29917.t1 | XP_011434510.2 | pfu_aug1.0_1966.1_29917.t1 |
| TRINITY_DN63933_c4_g2_i1 | -5.88 | 1.04E-03 |  |  |  |  |  |
| TRINITY_DN63964_c9_g1_i3 | -3.55 | 8.38E-04 |  |  | pfu_aug1.0_642.1_65556.t1 |  | pfu_aug1.0_642.1_65556.t1 |
| TRINITY_DN64220_c0_g1_i8 | -2.92 | 6.82E-03 | Q6ZSM3 | MOT12_HUMAN | pfu_aug1.0_3287.1_23283.t1 | XP_011436919.1 | Q6ZSM3 |
| TRINITY_DN64329_c0_g1_i1 | -1.06 | 2.90E-06 | Q09460 | PDFR1_CAEEL | pfu_aug1.0_11471.1_68338.t1 | XP_011432315.1 | pfu_aug1.0_11471.1_68338.t1 |
| TRINITY_DN64498_c0_g1_i3 | -3.45 | 2.25E-03 | H2A0N2 | NCP_PINMG | pfu_aug1.0_12300.1_32179.t1 |  | H2A0N2 |
| TRINITY_DN64532_c0_g1_i4 | -3.13 | 8.07E-03 |  |  | pfu_aug1.0_16145.1_25491.t1 |  | pfu_aug1.0_16145.1_25491.t1 |
| TRINITY_DN64726_c2_g1_i2 | -3.5 | 2.91E-04 |  |  | pfu_aug1.0_7764.1_60295.t1 | XP_011421065.1 | pfu_aug1.0_7764.1_60295.t1 |
| TRINITY_DN65030_c0_g1_i1 | -2.76 | 7.56E-04 |  |  | pfu_aug1.0_4701.1_08970.t1 |  | pfu_aug1.0_4701.1_08970.t1 |
| TRINITY_DN65245_c2_g1_i14 | -6.38 | 1.80E-04 |  |  |  |  |  |
| TRINITY_DN65245_c3_g1_i1 | -3.78 | 3.71E-12 | Q19673 | TYR3_CAEEL | pfu_aug1.0_14315.1_54050.t1 | XP_011429695.1 | XP_011429695.1 |
| TRINITY_DN65455_c1_g2_i1 | -1.35 | 8.59E-05 |  |  |  |  |  |
| TRINITY_DN65638_c0_g1_i1 | -5.51 | 4.40E-07 |  |  | pfu_aug1.0_40012.1_41153.t1 |  | pfu_aug1.0_40012.1_41153.t1 |
| TRINITY_DN65815_c0_g1_i6 | -5.21 | 5.38E-03 |  |  | pfu_aug1.0_7016.1_24128.t1 | XP_019924528.1 | XP_019924528.1 |
| TRINITY_DN65978_c0_g1_i1 | -5.9 | 2.46E-15 |  |  | pfu_aug1.0_346.1_15012.t1 |  | pfu_aug1.0_346.1_15012.t1 |
| TRINITY_DN65978_c0_g2_i3 | -4.54 | 6.11E-13 |  |  | pfu_aug1.0_346.1_15010.t1 | XP_019919272.1 | pfu_aug1.0_346.1_15010.t1 |
| TRINITY_DN66012_c5_g1_i1 | -3.61 | 1.35E-10 | Q7Z0T3 | TEMPT_APLCA | pfu_aug1.0_1127.1_65820.t1 | XP_011442116.1 | XP_011442116.1 |
| TRINITY_DN66044_c0_g2_i4 | -3.3 | 2.54E-04 | Q8NFD2 | ANKK1_HUMAN | pfu_aug1.0_6386.1_67430.t1 | XP_011413127.1 | XP_011413127.1 |
| TRINITY_DN66070_c3_g6_i1 | -2.23 | 9.72E-06 | P39876 | TIMP3_MOUSE | pfu_aug1.0_4074.1_59431.t1 | XP_011447814.1 | pfu_aug1.0_4074.1_59431.t1 |
| TRINITY_DN66196_c0_g2_i10 | -5.85 | 1.86E-07 |  |  |  |  |  |
| TRINITY_DN66196_c1_g1_i8 | -5.75 | 5.41E-04 |  |  |  |  |  |
| TRINITY_DN66196_c1_g2_i2 | -5.99 | 7.83E-10 |  |  |  |  |  |
| TRINITY_DN66282_c0_g1_i3 | -1.07 | 3.28E-03 | H2A0M0 | NRP_PINMG | pfu_aug1.0_1030.1_65747.t1 | XP_011452008.1 | pfu_aug1.0_1030.1_65747.t1 |
| TRINITY_DN66387_c6_g1_i1 | -4.9 | 7.01E-13 |  |  | pfu_aug1.0_48663.1_48682.t1 |  |  |
| TRINITY_DN66560_c0_g1_i7 | -6.26 | 1.07E-05 |  |  |  |  |  |
| TRINITY_DN66560_c0_g2_i3 | -4.68 | 8.16E-05 |  |  | pfu_aug1.0_2774.1_15943.t1 |  | pfu_aug1.0_2774.1_15943.t1 |
| TRINITY_DN66574_c1_g1_i2 | -4.24 | 2.40E-07 |  |  | pfu_aug1.0_28455.1_26424.t1 | XP_011419907.1 | pfu_aug1.0_28455.1_26424.t1 |
| TRINITY_DN66763_c6_g1_i1 | -7.47 | 6.82E-16 | H2A0L0 | TYRO1_PINMG | pfu_aug1.0_4.1_57868.t1 | XP_019928442.1 | pfu_aug1.0_4.1_57868.t1 |
| TRINITY_DN66822_c4_g3_i2 | -1.99 | 6.72E-05 | P11369 | LORF2_MOUSE | pfu_aug1.0_12090.1_10410.t1 | XP_019929360.1 | pfu_aug1.0_12090.1_10410.t1 |
| TRINITY_DN67340_c0_g1_i2 | 1.81 | 9.14E-04 |  |  | pfu_aug1.0_5615.1_59833.t1 | XP_011448183.1 | pfu_aug1.0_5615.1_59833.t1 |
| TRINITY_DN67393_c1_g1_i8 | -1.28 | 7.96E-03 | P14381 | YTX2_XENLA | pfu_aug1.0_8402.1_38747.t1 | XP_019927471.1 | pfu_aug1.0_8402.1_38747.t1 |
| TRINITY_DN67507_c1_g3_i1 | 4.6 | 6.23E-04 |  |  | pfu_aug1.0_17656.1_11055.t1 |  | pfu_aug1.0_17656.1_11055.t1 |
| TRINITY_DN67558_c2_g1_i1 | -2.92 | 8.37E-03 | Q8UWA5 | CAH2_TRIHK | pfu_aug1.0_22914.1_33303.t1 | XP_011435804.1 | XP_011435804.1 |
| TRINITY_DN67575_c0_g1_i5 | -4.9 | 9.84E-03 |  |  |  |  |  |
| TRINITY_DN67600_c0_g1_i2 | -2.05 | 4.85E-03 |  |  | pfu_aug1.0_1100.1_65803.t1 | XP_011430170.1 | pfu_aug1.0_1100.1_65803.t1 |
| TRINITY_DN67635_c0_g1_i3 | -7.73 | 9.33E-06 | O96790 | DPGN_DIPMA | pfu_aug1.0_8389.1_24384.t1 | XP_011449182.1 | pfu_aug1.0_8389.1_24384.t1 |
| TRINITY_DN67639_c0_g2_i2 | -5.82 | 2.71E-03 | P86950 | SLP2_PINMA |  |  | P86950 |
| TRINITY_DN67930_c2_g2_i3 | -5.53 | 3.26E-14 |  |  |  |  |  |
| TRINITY_DN67998_c1_g1_i3 | -7 | 1.74E-06 | Q9ULY5 | CLC4E_HUMAN | pfu_aug1.0_650.1_65564.t1 | XP_019920711.1 | XP_019920711.1 |
| TRINITY_DN68004_c7_g3_i1 | -1.39 | 8.76E-03 |  |  | pfu_aug1.0_79495.1_13214.t1 | XP_011421857.1 | pfu_aug1.0_79495.1_13214.t1 |
| TRINITY_DN68057_c0_g1_i2 | -2.85 | 4.28E-05 | Q9W332 | CUBN_DROME | pfu_aug1.0_1973.1_08191.t1 | XP_011437441.2 | pfu_aug1.0_1973.1_08191.t1 |
| TRINITY_DN68348_c0_g1_i1 | -4.07 | 4.61E-03 |  |  | pfu_aug1.0_20147.1_61975.t1 | XP_011447607.1 | XP_011447607.1 |
| TRINITY_DN68384_c0_g1_i1 | -1.51 | 2.11E-04 |  |  | pfu_aug1.0_4930.1_52401.t1 |  | pfu_aug1.0_4930.1_52401.t1 |
| TRINITY_DN68823_c7_g2_i1 | -2.63 | 7.68E-03 | O08746 | MATN2_MOUSE | pfu_aug1.0_759.1_15228.t1 | XP_011415576.1 | pfu_aug1.0_759.1_15228.t1 |
| TRINITY_DN68917_c0_g3_i7 | -5.28 | 2.46E-03 | H2A0K6 | VRP_PINMG | pfu_aug1.0_89.1_57967.t1 | XP_011421288.1 | H2A0K6 |
| TRINITY_DN68963_c0_g1_i4 | -4.38 | 1.21E-11 |  |  |  |  |  |
| TRINITY_DN69086_c1_g1_i1 | -2.46 | 1.56E-03 |  |  |  |  |  |
| TRINITY_DN69254_c0_g1_i3 | -2.35 | 8.65E-03 |  |  | pfu_aug1.0_23958.1_62267.t1 | XP_011418688.1 | pfu_aug1.0_23958.1_62267.t1 |
| TRINITY_DN69360_c0_g1_i4 | -6.17 | 1.02E-20 |  |  |  |  |  |
| TRINITY_DN69380_c0_g1_i4 | -1.48 | 1.46E-03 | Q5E9U6 | WNT16_BOVIN | pfu_aug1.0_8897.1_53252.t1 | XP_011423677.1 | XP_011423677.1 |
| TRINITY_DN69386_c0_g1_i7 | -7.37 | 9.42E-12 |  |  | pfu_aug1.0_251170.1_35896.t1 | XP_011452816.1 | XP_011452816.1 |
| TRINITY_DN69472_c2_g1_i2 | -3.14 | 1.41E-04 | P13670 | CHB_VIBHA | pfu_aug1.0_6745.1_67527.t1 | XP_011422975.1 | pfu_aug1.0_6745.1_67527.t1 |
| TRINITY_DN69810_c1_g1_i8 | -1.76 | 1.73E-04 | Q8K385 | FRRS1_MOUSE | pfu_aug1.0_1539.1_08010.t1 | XP_011432083.1 | pfu_aug1.0_1539.1_08010.t1 |
| TRINITY_DN69959_c1_g1_i3 | -1.89 | 3.94E-04 |  |  | pfu_aug1.0_49139.1_12675.t1 | XP_019928755.1 | pfu_aug1.0_49139.1_12675.t1 |
| TRINITY_DN70506_c2_g1_i1 | -4.63 | 8.53E-03 | H2A0L9 | SGYQP_PINMG | pfu_aug1.0_17386.1_69084.t1 |  | H2A0L9 |
| TRINITY_DN70850_c0_g1_i2 | -2.54 | 1.25E-03 | Q7M4T0 | NEG1_NEUCR | pfu_aug1.0_12836.1_53866.t1 | XP_011456351.1 | pfu_aug1.0_12836.1_53866.t1 |
| TRINITY_DN71057_c1_g1_i3 | -2.45 | 1.77E-15 |  |  | pfu_aug1.0_86976.1_20664.t1 |  | pfu_aug1.0_86976.1_20664.t1 |
| TRINITY_DN71268_c0_g5_i1 | -2.64 | 2.28E-03 |  |  |  | XP_011412992.1 | XP_011412992.1 |
| TRINITY_DN71304_c1_g1_i1 | -5.87 | 4.19E-09 | H2A0L6 | HEX_PINMG | pfu_aug1.0_5129.1_45120.t1 | XP_011422976.1 | H2A0L6 |
| TRINITY_DN71462_c0_g1_i1 | -3.03 | 1.24E-04 |  |  | pfu_aug1.0_10577.1_53557.t1 |  | pfu_aug1.0_10577.1_53557.t1 |
| TRINITY_DN71751_c1_g1_i4 | -2.61 | 4.36E-03 | Q20930 | MIG17_CAEEL | pfu_aug1.0_1843.1_37146.t1 | XP_011438907.1 | XP_011438907.1 |
| TRINITY_DN71896_c4_g1_i4 | 2.69 | 5.41E-04 |  |  |  |  |  |
| TRINITY_DN71916_c0_g1_i6 | -5.35 | 6.82E-05 |  |  |  |  |  |
| TRINITY_DN72345_c0_g4_i1 | -4.11 | 6.11E-16 | H2A0N1 | KCP2_PINMG | pfu_aug1.0_1882.1_08167.t1 | XP_011446794.1 | H2A0N1 |
| TRINITY_DN72578_c0_g1_i1 | -3.44 | 6.92E-03 | P31429 | DPEP1_RABIT | pfu_aug1.0_12595.1_46534.t1 | XP_011433867.1 | XP_011433867.1 |
| TRINITY_DN72684_c1_g1_i5 | -1.86 | 3.39E-06 | A6QLQ8 | ENDOU_BOVIN | pfu_aug1.0_93349.1_42297.t1 | XP_011428046.1 | XP_011428046.1 |
| TRINITY_DN72913_c0_g1_i1 | -4.96 | 4.96E-07 | H2A0M3 | AMO_PINMG | pfu_aug1.0_1052.1_07781.t1 | XP_011438553.1 | H2A0M3 |
| TRINITY_DN73050_c0_g1_i1 | -6.69 | 1.99E-05 | P86730 | PWAP_HALAI | pfu_aug1.0_102.1_29088.t1 | XP_011418342.1 | pfu_aug1.0_102.1_29088.t1 |
| TRINITY_DN73280_c0_g2_i11 | -6.82 | 1.73E-14 |  |  |  |  |  |
| TRINITY_DN73622_c0_g1_i3 | -1.25 | 9.03E-03 |  |  | pfu_aug1.0_112950.1_57042.t1 | XP_011413197.1 | pfu_aug1.0_112950.1_57042.t1 |
| TRINITY_DN73716_c1_g1_i2 | -4.98 | 9.56E-05 | H2A0L5 | CHI2_PINMG | pfu_aug1.0_10761.1_31980.t1 | NP_001295799.1 | H2A0L5 |
| TRINITY_DN73890_c1_g1_i1 | -1.11 | 9.31E-03 |  |  | pfu_aug1.0_230249.1_14267.t1 | XP_019923133.1 | pfu_aug1.0_230249.1_14267.t1 |
| TRINITY_DN73922_c1_g3_i1 | -3.23 | 2.40E-09 |  |  |  |  |  |
| TRINITY_DN74460_c1_g3_i5 | -1.46 | 5.53E-07 |  |  | pfu_aug1.0_3739.1_37682.t1 |  | pfu_aug1.0_3739.1_37682.t1 |
| TRINITY_DN74559_c0_g1_i1 | -2.72 | 4.73E-03 | Q96JK4 | HIPL1_HUMAN | pfu_aug1.0_14711.1_54097.t1 | XP_019925916.1 | XP_019925916.1 |
| TRINITY_DN74612_c0_g1_i1 | -1.62 | 3.66E-03 | P79102 | CP3AS_BOVIN | pfu_aug1.0_9405.1_17476.t1 | XP_019930648.1 | pfu_aug1.0_9405.1_17476.t1 |
| TRINITY_DN74652_c2_g1_i3 | -2.78 | 6.72E-04 |  |  | pfu_aug1.0_12591.1_03255.t1 |  |  |
| TRINITY_DN74750_c5_g1_i12 | -3.63 | 3.19E-09 |  |  | pfu_aug1.0_3154.1_30351.t1 |  | pfu_aug1.0_3154.1_30351.t1 |
| TRINITY_DN75091_c1_g1_i3 | -5.81 | 3.29E-09 |  |  | pfu_aug1.0_14726.1_46800.t1 | XP_011448738.1 | pfu_aug1.0_14726.1_46800.t1 |
| TRINITY_DN75124_c2_g1_i2 | -2.84 | 8.95E-03 | Q9VCA2 | ORCT_DROME | pfu_aug1.0_298.1_22126.t1 | XP_011433969.1 | XP_011433969.1 |
| TRINITY_DN75500_c0_g1_i2 | -2.41 | 2.11E-04 | H2A0L7 | FND1_PINMG | pfu_aug1.0_6844.1_09446.t1 | XP_011414106.1 | H2A0L7 |
| TRINITY_DN75536_c0_g1_i1 | -3.43 | 7.43E-04 |  |  | pfu_aug1.0_2225.1_44288.t1 |  | pfu_aug1.0_2225.1_44288.t1 |
| TRINITY_DN75550_c0_g1_i4 | -4.12 | 1.74E-13 |  |  | pfu_aug1.0_22730.1_18891.t1 | XP_019926576.1 | pfu_aug1.0_22730.1_18891.t1 |
| TRINITY_DN75690_c3_g3_i1 | -4.64 | 1.47E-12 | H2A0L2 | ELDP1_PINMG | pfu_aug1.0_1213.1_29620.t1 | XP_011437437.1 | H2A0L2 |
| TRINITY_DN75844_c0_g1_i2 | -6.57 | 3.40E-09 | P86952 | TYRO_PINMA | pfu_aug1.0_130908.1_28331.t1 | NP_001292226.1 | NP_001292226.1 |
| TRINITY_DN76046_c0_g3_i5 | -4.11 | 2.32E-04 | Q03610 | YN81_CAEEL | pfu_aug1.0_13299.1_03339.t1 | XP_011414762.1 | pfu_aug1.0_13299.1_03339.t1 |
| TRINITY_DN76826_c0_g1_i2 | -4.81 | 7.50E-03 |  |  | pfu_aug1.0_3525.1_23341.t1 |  | pfu_aug1.0_3525.1_23341.t1 |
| TRINITY_DN76913_c1_g1_i3 | -5.22 | 2.57E-14 |  |  | pfu_aug1.0_15095.1_10774.t1 | XP_011432382.1 | pfu_aug1.0_15095.1_10774.t1 |
| TRINITY_DN76993_c5_g5_i1 | 1.06 | 7.40E-03 |  |  | pfu_aug1.0_48372.1_12654.t1 | XP_019924775.1 | pfu_aug1.0_48372.1_12654.t1 |
| TRINITY_DN77105_c0_g1_i2 | -4.84 | 3.18E-05 |  |  | pfu_aug1.0_10419.1_46204.t1 |  | pfu_aug1.0_10419.1_46204.t1 |
| TRINITY_DN77333_c0_g1_i4 | -3.61 | 1.58E-06 | P69525 | TMPS9_MOUSE | pfu_aug1.0_21886.1_40322.t1 | XP_019927952.1 | pfu_aug1.0_21886.1_40322.t1 |
| TRINITY_DN77372_c1_g1_i7 | -3.26 | 8.81E-07 | P34269 | TYR1_CAEEL | pfu_aug1.0_13287.1_68623.t1 | XP_011416062.1 | pfu_aug1.0_13287.1_68623.t1 |
| TRINITY_DN77400_c0_g1_i1 | -3.08 | 3.29E-04 |  |  |  |  |  |
| TRINITY_DN77456_c3_g2_i5 | -6.1 | 3.90E-13 |  |  | pfu_aug1.0_14315.1_54050.t1 | XP_011429695.1 | pfu_aug1.0_14315.1_54050.t1 |
| TRINITY_DN77525_c0_g1_i1 | -3.08 | 3.39E-03 |  |  | pfu_aug1.0_258385.1_07007.t1 | | pfu_aug1.0_258385.1_07007.t1 |
| TRINITY_DN77579_c1_g1_i8 | -5.18 | 5.14E-04 | Q99PG2 | OGFR_MOUSE | pfu_aug1.0_70381.1_56474.t1 | XP_011420326.1 | pfu_aug1.0_70381.1_56474.t1 |
| TRINITY_DN77617_c2_g1_i2 | -4.69 | 2.56E-09 |  |  |  |  |  |
| TRINITY_DN77651_c2_g1_i1 | -3.21 | 3.40E-05 |  |  | pfu_aug1.0_7162.1_67645.t1 |  | pfu_aug1.0_7162.1_67645.t1 |
| TRINITY_DN77978_c1_g1_i4 | -6.54 | 1.89E-13 |  |  | pfu_aug1.0_1060.1_65769.t1 | XP_011452815.1 | pfu_aug1.0_1060.1_65769.t1 |
| TRINITY_DN78009_c2_g1_i1 | -3.27 | 7.76E-12 |  |  | pfu_aug1.0_1547.1_15520.t1 | XP_011449235.1 | XP_011449235.1 |
| TRINITY_DN78068_c0_g1_i9 | -2.1 | 5.03E-10 |  |  |  |  |  |
| TRINITY_DN78116_c0_g2_i1 | 2.55 | 7.72E-04 |  |  | pfu_aug1.0_12874.1_03293.t1 |  | pfu_aug1.0_12874.1_03293.t1 |
| TRINITY_DN78183_c0_g1_i1 | -5.91 | 1.49E-07 | Q01603 | PERO_DROME | pfu_aug1.0_19559.1_61927.t1 | XP_011434623.1 | pfu_aug1.0_19559.1_61927.t1 |
| TRINITY_DN78465_c0_g1_i1 | -2.07 | 2.08E-04 | P86789 | GIGA6_CRAGI | pfu_aug1.0_102009.1_13605.t1 | XP_011449645.1 | pfu_aug1.0_102009.1_13605.t1 |
| TRINITY_DN78548_c0_g1_i8 | -4.85 | 7.75E-07 |  |  | pfu_aug1.0_3068.1_23160.t1 | XP_011412527.1 | pfu_aug1.0_3068.1_23160.t1 |
| TRINITY_DN78585_c0_g1_i7 | -4.44 | 3.18E-14 |  |  |  |  |  |
| TRINITY_DN78700_c1_g1_i1 | -4.35 | 8.09E-05 | P86962 | USP2_PINMA | pfu_aug1.0_43555.1_55905.t1 |  | P86962 |
| TRINITY_DN78777_c1_g4_i1 | -1.58 | 2.02E-03 |  |  | pfu_aug1.0_16069.1_39778.t1 | XP_019929791.1 | pfu_aug1.0_16069.1_39778.t1 |
| TRINITY_DN78991_c1_g1_i2 | -4.86 | 1.23E-16 |  |  | pfu_aug1.0_14699.1_32469.t1 |  | pfu_aug1.0_14699.1_32469.t1 |
| TRINITY_DN79528_c0_g1_i1 | -3.33 | 2.17E-04 |  |  | pfu_aug1.0_5820.1_52622.t1 | XP_011426878.1 | pfu_aug1.0_5820.1_52622.t1 |
| TRINITY_DN79528_c0_g2_i3 | -5.33 | 1.74E-13 |  |  | pfu_aug1.0_5820.1_52622.t1 | XP_019918429.1 | pfu_aug1.0_5820.1_52622.t1 |
| TRINITY_DN79672_c0_g2_i1 | -1.76 | 7.14E-04 | Q24372 | LACH_DROME | pfu_aug1.0_9454.1_46039.t1 | XP_011431452.1 | pfu_aug1.0_9454.1_46039.t1 |
| TRINITY_DN79733_c2_g1_i15 | -2.95 | 2.47E-06 | Q6GM59 | MOT12_XENLA | pfu_aug1.0_12502.1_32215.t1 | XP_019919099.1 | Q6GM59 |
| TRINITY_DN79733_c2_g3_i1 | -3.14 | 3.25E-09 | O15375 | MOT6_HUMAN | pfu_aug1.0_860.1_51092.t1 | XP_011436919.1 | pfu_aug1.0_860.1_51092.t1 |
| TRINITY_DN79948_c3_g2_i2 | -4.55 | 6.19E-06 |  |  | pfu_aug1.0_16145.1_25491.t1 |  | pfu_aug1.0_16145.1_25491.t1 |
| TRINITY_DN80061_c0_g2_i4 | -3.69 | 6.15E-03 | P56730 | NETR_HUMAN | pfu_aug1.0_21886.1_40322.t1 | XP_011445228.1 | pfu_aug1.0_21886.1_40322.t1 |
| TRINITY_DN80193_c1_g1_i6 | -1.49 | 5.39E-06 | P26652 | TIMP3_CHICK | pfu_aug1.0_24427.1_26163.t1 | XP_011445255.1 | XP_011445255.1 |
| TRINITY_DN80220_c1_g3_i1 | -5.02 | 3.54E-07 |  |  | pfu_aug1.0_10222.1_60749.t1 |  | pfu_aug1.0_10222.1_60749.t1 |
| TRINITY_DN80736_c0_g1_i1 | -4.2 | 1.38E-08 |  |  | pfu_aug1.0_6087.1_09272.t1 | XP_011424911.1 | pfu_aug1.0_6087.1_09272.t1 |
| TRINITY_DN80736_c0_g2_i1 | -3.75 | 1.66E-08 | P36633 | AOC1_RAT | pfu_aug1.0_6087.1_09272.t1 | XP_011424907.2 | XP_011424907.2 |
| TRINITY_DN80822_c0_g1_i16 | -3.53 | 5.45E-11 |  |  | pfu_aug1.0_17466.1_03830.t1 |  | pfu_aug1.0_17466.1_03830.t1 |
| TRINITY_DN80898_c1_g2_i4 | -1.85 | 3.73E-04 | Q20930 | MIG17_CAEEL | pfu_aug1.0_14511.1_54062.t1 | XP_019926910.1 | pfu_aug1.0_14511.1_54062.t1 |
| TRINITY_DN81016_c1_g2_i3 | -1.75 | 2.84E-04 | Q02844 | TRYB1_MOUSE | pfu_aug1.0_2803.1_30249.t1 | XP_011419617.1 | XP_011419617.1 |
| TRINITY_DN81016_c1_g3_i2 | -1.72 | 2.35E-03 | Q640N1 | AEBP1_MOUSE | pfu_aug1.0_3370.1_59213.t1 | XP_011419616.1 | XP_011419616.1 |
| TRINITY_DN81089_c0_g1_i6 | -2.44 | 5.03E-10 |  |  |  |  |  |
| TRINITY_DN81129_c0_g1_i5 | -1.29 | 3.89E-04 |  |  | pfu_aug1.0_46712.1_12616.t1 | XP_011455168.1 | XP_011455168.1 |
| TRINITY_DN81129_c0_g4_i1 | -1.4 | 4.85E-05 |  |  | pfu_aug1.0_8069.1_67796.t1 | XP_011455168.1 | pfu_aug1.0_8069.1_67796.t1 |
| TRINITY_DN81393_c1_g1_i3 | -2.07 | 8.00E-03 | P00766 | CTRA_BOVIN | pfu_aug1.0_17175.1_61690.t1 | XP_011445228.1 | pfu_aug1.0_17175.1_61690.t1 |
| TRINITY_DN81480_c0_g1_i1 | -2.29 | 1.86E-03 | O95238 | SPDEF_HUMAN | pfu_aug1.0_5375.1_30945.t2 | XP_011443811.1 | pfu_aug1.0_5375.1_30945.t2 |
| TRINITY_DN81587_c0_g1_i6 | -1.78 | 4.68E-06 |  |  |  |  |  |
| TRINITY_DN81705_c0_g1_i11 | -6.41 | 2.99E-05 | H2A0K8 | SLP1_PINMG | pfu_aug1.0_24266.1_33421.t1 |  | H2A0K8 |
| TRINITY_DN81705_c0_g3_i1 | -6.35 | 2.14E-05 | P86950 | SLP2_PINMA |  |  | P86950 |
| TRINITY_DN82426_c2_g1_i6 | -1.53 | 9.04E-04 | P56405 | AQP8_RAT | pfu_aug1.0_33748.1_55478.t1 | XP_011436665.1 | XP_011436665.1 |
| TRINITY_DN82475_c1_g1_i2 | 1.85 | 1.09E-03 | P21328 | RTJK_DROME | pfu_aug1.0_811.1_58262.t1 | XP_019922330.1 | XP_019922330.1 |
| TRINITY_DN82732_c1_g1_i1 | -5.6 | 1.24E-04 |  |  |  |  |  |
| TRINITY_DN82763_c3_g2_i1 | -1.28 | 4.04E-06 |  |  | pfu_aug1.0_498335.1_36151.t1 | XP_011441107.1 | |
| TRINITY_DN82846_c0_g1_i1 | -2.27 | 8.84E-05 |  |  | pfu_aug1.0_8184.1_60379.t1 | XP_019923112.1 | pfu_aug1.0_8184.1_60379.t1 |
| TRINITY_DN82975_c0_g1_i2 | -4.95 | 5.03E-10 | P86950 | SLP2_PINMA | pfu_aug1.0_14895.1_10758.t1 |  | pfu_aug1.0_14895.1_10758.t1 |
| TRINITY_DN82987_c1_g1_i1 | -3.88 | 5.05E-04 | H2A0L1 | TYRO2_PINMG | pfu_aug1.0_16905.1_25558.t1 | XP_011416064.1 | H2A0L1 |
| TRINITY_DN83064_c1_g1_i9 | -4.15 | 7.14E-04 | H2A0L7 | FND1_PINMG | pfu_aug1.0_6844.1_09446.t1 | XP_011414106.1 | H2A0L7 |
| TRINITY_DN83138_c1_g2_i2 | -3.76 | 7.46E-05 | H2A0N1 | KCP2_PINMG | pfu_aug1.0_1882.1_08167.t1 | XP_011446794.1 | H2A0N1 |
| TRINITY_DN83312_c1_g1_i3 | -4.86 | 1.02E-06 | H2A0M7 | PLSP_PINMG | pfu_aug1.0_1885.1_51458.t1 | XP_019921592.1 | H2A0M7 |
| TRINITY_DN83438_c1_g1_i1 | -7.71 | 7.13E-09 |  |  | pfu_aug1.0_31528.1_04778.t1 |  | pfu_aug1.0_31528.1_04778.t1 |
| TRINITY_DN83516_c0_g1_i2 | -4.45 | 2.64E-03 |  |  |  |  |  |
| TRINITY_DN83516_c0_g2_i4 | -3.92 | 6.34E-03 |  |  | pfu_aug1.0_2774.1_15943.t1 |  | pfu_aug1.0_2774.1_15943.t1 |
| TRINITY_DN83632_c2_g1_i1 | -3.35 | 6.54E-19 | Q98ST7 | MOXD1_CHICK | pfu_aug1.0_1065.1_36810.t1 | XP_011418505.1 | pfu_aug1.0_1065.1_36810.t1 |
| TRINITY_DN83738_c2_g1_i1 | -4.3 | 7.58E-09 |  |  | pfu_aug1.0_3972.1_16226.t1 | XP_011452009.1 | pfu_aug1.0_3972.1_16226.t1 |
| TRINITY_DN83738_c2_g3_i1 | -4.49 | 3.08E-06 |  |  | pfu_aug1.0_3972.1_16226.t1 | XP_011452009.1 | pfu_aug1.0_3972.1_16226.t1 |
| TRINITY_DN83829_c0_g1_i3 | -4.6 | 2.85E-07 | Q8Q0U0 | Y045_METMA | pfu_aug1.0_2.1_14613.t1 | XP_011445311.1 | XP_011445311.1 |
| TRINITY_DN84180_c0_g1_i1 | -2.69 | 3.57E-03 | Q80VI1 | TRI56_MOUSE | pfu_aug1.0_4178.1_44882.t1 | XP_011429480.1 | pfu_aug1.0_4178.1_44882.t1 |
| TRINITY_DN84180_c0_g2_i2 | -3.25 | 1.50E-08 |  |  | pfu_aug1.0_4178.1_44882.t1 | XP_011429480.1 | pfu_aug1.0_4178.1_44882.t1 |
| TRINITY_DN84370_c4_g1_i1 | -3.07 | 1.68E-04 | H2A0M4 | USP1_PINMG | pfu_aug1.0_12300.1_32179.t1 | XP_011456443.1 | H2A0M4 |
| TRINITY_DN84435_c0_g2_i3 | -2.62 | 2.69E-04 | P98157 | LRP1_CHICK | pfu_aug1.0_5759.1_09217.t1 | XP_011430016.1 | XP_011430016.1 |
| TRINITY_DN84442_c1_g2_i2 | -1.68 | 1.37E-03 |  |  | pfu_aug1.0_4889.1_09005.t1 |  | pfu_aug1.0_4889.1_09005.t1 |
| TRINITY_DN85066_c1_g1_i5 | -2.81 | 1.05E-08 | H2A0M7 | PLSP_PINMG | pfu_aug1.0_4298.1_08859.t1 | XP_019921592.1 | XP_019921592.1 |
| TRINITY_DN85198_c1_g1_i3 | -2.66 | 2.15E-03 | Q7QH73 | PERC_ANOGA | pfu_aug1.0_3964.1_08779.t1 | XP_019920121.1 | pfu_aug1.0_3964.1_08779.t1 |
| TRINITY_DN85487_c1_g1_i2 | -1.45 | 5.97E-11 |  |  | pfu_aug1.0_163.1_36324.t1 |  | pfu_aug1.0_163.1_36324.t1 |
| TRINITY_DN85487_c1_g3_i9 | -1.21 | 2.78E-06 | A1BQQ5 | MR30_CONMR | pfu_aug1.0_163.1_36327.t1 | XP_011449942.1 | XP_011449942.1 |
| TRINITY_DN85504_c1_g2_i2 | -1.34 | 1.99E-08 | Q0EEE2 | PTHD3_MOUSE | pfu_aug1.0_2100.1_22869.t1 | XP_011442864.1 | pfu_aug1.0_2100.1_22869.t1 |
| TRINITY_DN85519_c0_g2_i2 | -2.7 | 2.14E-05 |  |  | pfu_aug1.0_3110.1_01338.t1 |  | pfu_aug1.0_3110.1_01338.t1 |
| TRINITY_DN85595_c0_g2_i4 | 2.13 | 6.05E-03 | P10401 | POLY_DROME | pfu_aug1.0_5819.1_02075.t1 | XP_019920316.1 | pfu_aug1.0_5819.1_02075.t1 |
| TRINITY_DN85694_c0_g1_i7 | -3.41 | 1.53E-07 | H2A0L4 | CHI1_PINMG | pfu_aug1.0_14887.1_32490.t1 | NP_001292252.1 | pfu_aug1.0_14887.1_32490.t1 |
| TRINITY_DN85773_c1_g1_i9 | -2.51 | 2.17E-04 | Q7D513 | EGTB_MYCTO | pfu_aug1.0_35351.1_48195.t1 | XP_011413777.1 | XP_011413777.1 |
| TRINITY_DN86318_c0_g1_i4 | -3.95 | 1.04E-03 |  |  | pfu_aug1.0_4099.1_08806.t1 |  | pfu_aug1.0_4099.1_08806.t1 |
| TRINITY_DN86362_c2_g3_i2 | -1.18 | 1.20E-03 | Q9GKY0 | FSTL1_MACFA | pfu_aug1.0_2012.1_22828.t1 | XP_011435865.1 | pfu_aug1.0_2012.1_22828.t1 |
| TRINITY_DN86425_c1_g1_i1 | -2.44 | 1.26E-05 | H2A0L1 | TYRO2_PINMG | pfu_aug1.0_123.1_50650.t1 | XP_011413535.1 | pfu_aug1.0_123.1_50650.t1 |
| TRINITY_DN86589_c4_g4_i4 | -1.82 | 2.91E-03 | H2A0L8 | FND2_PINMG | pfu_aug1.0_6844.1_09446.t1 | XP_011414106.1 | H2A0L8 |
| TRINITY_DN86664_c6_g1_i3 | -5.55 | 7.83E-10 |  |  | pfu_aug1.0_507.1_36562.t1 |  | pfu_aug1.0_507.1_36562.t1 |
| TRINITY_DN86829_c2_g1_i1 | -3.45 | 3.47E-08 | Q5R9A7 | GP155_PONAB | pfu_aug1.0_648.1_15168.t1 | XP_019928755.1 | XP_019928755.1 |
| TRINITY_DN86829_c2_g2_i1 | -3.08 | 8.87E-07 | Q5R9A7 | GP155_PONAB | pfu_aug1.0_49139.1_12675.t1 | XP_019928755.1 | pfu_aug1.0_49139.1_12675.t1 |
| TRINITY_DN87184_c6_g1_i1 | -1.46 | 3.36E-05 | O88281 | MEGF6_RAT | pfu_aug1.0_20326.1_40226.t1 | XP_011429547.1 | XP_011429547.1 |
| TRINITY_DN87258_c5_g3_i1 | -4.21 | 1.00E-13 | A4QP81 | FRRS1_DANRE | pfu_aug1.0_5514.1_23808.t1 | XP_011434249.1 | XP_011434249.1 |
| TRINITY_DN87296_c4_g1_i2 | -3.08 | 1.75E-11 |  |  | pfu_aug1.0_6700.1_02292.t1 | XP_011449016.1 | pfu_aug1.0_6700.1_02292.t1 |
| TRINITY_DN87297_c3_g1_i4 | -4.21 | 5.15E-04 |  |  | pfu_aug1.0_164724.1_21334.t1 | | pfu_aug1.0_164724.1_21334.t1 |
| TRINITY_DN87298_c1_g1_i6 | -1.12 | 4.94E-03 |  |  | pfu_aug1.0_248.1_14931.t1 | XP_011424388.1 | pfu_aug1.0_248.1_14931.t1 |
| TRINITY_DN88070_c3_g1_i1 | -1.19 | 3.55E-04 | Q8IRI6 | GTR1_DROME | pfu_aug1.0_805.1_58255.t1 | XP_019926257.1 | XP_019926257.1 |
| TRINITY_DN88445_c4_g1_i3 | -4.33 | 5.72E-04 |  |  | pfu_aug1.0_2774.1_15943.t1 |  | pfu_aug1.0_2774.1_15943.t1 |
| TRINITY_DN88452_c5_g1_i14 | -5.05 | 3.39E-03 |  |  |  |  |  |
| TRINITY_DN96791_c0_g1_i1 | -3.71 | 3.99E-03 |  |  | pfu_aug1.0_16176.1_10901.t1 | XP_011435189.2 | pfu_aug1.0_16176.1_10901.t1 |
| TRINITY_DN98546_c0_g1_i1 | -2.37 | 3.97E-03 |  |  |  |  |  |

Table S3

Table S3: Set of forward and reverse primers used for the biomineralization gene expression (real-time PCR) analysis in *Pinctada margaritifera*.

| **Fonction** | **Gene** | **Sequences** | **Genbank** | **References** |
| --- | --- | --- | --- | --- |
| housekeeping | EF | F- CCACGAGTCCTTACCAGAGG  R- TGGATCACTTTTGCTGTCTCC |  |  |
| housekeeping | GADPH | F - AGGCTTGATGACCACTGTCC  R - AGCCATTCCCGTCAACTTC |  |  |
| Nacreous | PIF177 | F - AGATTGAGGGCATAGCATGG  R - TGAGGCCGACTTTCTTGG | HE610401 | [1] |
| Prismatic | Shematrin9 | F - TGGAAGCAGTTTGACAGGTG  R - ATACCAGGGCCGTAAATGC | EF160120 |  |
| Prismatic | Aspein | F - TGAAGGGGATAGCCATTCTTC  R - ACTCGGTTCGGAAACAACTG | SRX022139 | [2, 3] |
| Prismatic | MP10 | F - GCCCGTCCACAGAACTAGAG  R - GATGAGGCACGTCTTTGACC | HE610374 | [4] |

References

1. Joubert C, Linard C, Le Moullac G, Soyez C, Saulnier D, Teaniniuraitemoana V, et al. Temperature and food influence shell growth and mantle gene expression of shell matrix proteins in the pearl oyster *Pinctada margaritifera*. PLoS ONE. 2014;9:e103944.

2. Isowa Y, Sarashina I, Setiamarga DHE, Endo K. A comparative study of the shell matrix protein aspein in Pterioid bivalves. J Mol Evol. 2012;75:11–8.

3. Tsukamoto D, Sarashina I, Endo K. Structure and expression of an unusually acidic matrix protein of pearl oyster shells. Biochem Biophys Res Commun. 2004;320:1175–80.

4. Joubert C, Piquemal D, Marie B, Manchon L, Pierrat F, Zanella-Cléon I, et al. Transcriptome and proteome analysis of *Pinctada margaritifera* calcifying mantle and shell: focus on biomineralization. BMC Genomics. 2010;11:613.
